# Comparison of CPI and GAP models in patients with idiopathic pulmonary fibrosis: a nationwide cohort study

**DOI:** 10.1038/s41598-018-23073-3

**Published:** 2018-03-19

**Authors:** Sang Hoon Lee, Jong Sun Park, Song Yee Kim, Dong Soon Kim, Young Whan Kim, Man Pyo Chung, Soo Taek Uh, Choon Sik Park, Sung Woo Park, Sung Hwan Jeong, Yong Bum Park, Hong Lyeol Lee, Jong Wook Shin, Eun Joo Lee, Jin Hwa Lee, Yangin Jegal, Hyun Kyung Lee, Yong Hyun Kim, Jin Woo Song, Moo Suk Park

**Affiliations:** 1 Department of Internal Medicine, Seoul National University College of Medicine, Seoul National University Bundang Hospital, 166, Gumi-ro, Bundang-gu, Seongnam-si, Gyeonggi-do 13620 Republic of Korea; 2 Department of Internal Medicine, Severance Hospital, Institute of Chest Diseases, Yonsei University College of Medicine, 50-1, Yonsei-ro, Seodaemun-gu, Seoul, 03772 Republic of Korea; 3 Division of Pulmonary and Critical Care Medicine, University of Ulsan College of Medicine, Asan Medical Center, 88, Olympic-ro 43-gil, Songpa-gu, Seoul, 05505 Republic of Korea; 4 Division of Pulmonary and Critical Care Medicine, Department of Internal Medicine and Lung Institute, Seoul National University College of Medicine, 101, Daehak-ro Jongno-gu, Seoul, 03080 Republic of Korea; 5 Division of Pulmonary and Critical Care Medicine, Samsung Medical Center, Sungkyunkwan University School of Medicine, 81 Irwon-ro Gangnam-gu, Seoul, 06351 Korea; 6 Division of Allergy and Respiratory Medicine, Department of Internal Medicine, Soonchunhyang University Seoul Hospital, 59, Daesagwan-ro, Yongsan-gu, Seoul, 04401 Republic of Korea; 7 Division of Allergy and Respiratory Medicine, Department of Internal Medicine, Soonchunhyang University Bucheon Hospital, 170 Jornaru-ro, Wonmi-gu, Bucheon, Gyeonggi-do 14584 Republic of Korea; 8 Division of Pulmonology, Department of Internal Medicine, Gachon University Gil Medical Center, 14, Namdong-daero 774beon-gil, Namdong-gu, Incheon, 21565 Korea; 9 Division of Pulmonary, Allergy & Critical Care Medicine, Department of Internal Medicine, Hallym University Kangdong Sacred Heart Hospital, 445, Gil-dong, Gangdong-gu, Seoul, 05355 Korea; 10 Department of Internal Medicine, Inha University Hospital, 27, Inhang-ro, Jung-gu, Incheon, 22332 Korea; 11 Division of Pulmonary Medicine, Department of Internal medicine, Chung Ang University College of Medicine, 102, Heukseok-ro, Dongjak-gu, Seoul, 06973 Korea; 12 Division of Respiratory and Critical Care Medicine, Department of Internal Medicine, Korea University Anam Hospital, Korea University College of Medicine, 73 Inchon-ro, Seongbuk-gu, Seoul, 02841 Korea; 13 Department of Internal Medicine, Ewha Womans University School of Medicine, Ewha Medical Research Institute, 1071, Anyangcheon-ro, Yangcheon-gu, Seoul, 07985 Korea; 14 Division of Pulmonary Medicine, Department of Internal Medicine, Ulsan University Hospital, University of Ulsan College of Medicine, 877, Bangeojinsunhwando-ro, Dong-gu, Ulsan, 44055 Korea; 15 Division of Critical Care and Pulmonary Medicine, Department of Internal Medicine, Inje University Pusan Paik Hospital, Bokji-ro 75, Busangjin-gu, Busan, 47392 Korea; 16 Division of Allergy and Pulmonology, Department of Internal Medicine, Bucheon St. Mary’s Hospital, The Catholic University of Korea School of Medicine, 327, Sosa-ro, Wonmi-gu, Bucheon-si, Gyeonggi-do 14647 Korea

## Abstract

The clinical course of idiopathic pulmonary fibrosis (IPF) is difficult to predict, partly owing to its heterogeneity. Composite physiologic index (CPI) and gender-age-physiology (GAP) models are easy-to-use predictors of IPF progression. This study aimed to compare the predictive values of these two models. From 2003 to 2007, the Korean Interstitial Lung Disease (ILD) Study Group surveyed ILD patients using the 2002 ATS/ERS criteria. A total of 832 patients with IPF were enrolled in this study. CPI was calculated as follows: 91.0 − (0.65 × %DL_CO_) − [0.53 × %FVC + [0.34 × %FEV_1_. GAP stage was calculated based on gender (0–1 points), age (0–2 points), and two physiologic lung function parameters (0–5 points). The two models had similar significant predictive values for patients with IPF (p < 0.001). The area under the curve (AUC) was higher for CPI than GAP for prediction of 1-, 2-, and 3-year mortality in this study. The AUC was higher for surgically diagnosed IPF patients than for clinically diagnosed patients. However, neither CPI nor GAP yielded good predictions of outcomes; the AUC was approximately 0.61~0.65. Although both CPI and GAP stage are significantly useful predictors for IPF, they have limited capability to accurately predict outcomes.

## Introduction

Idiopathic pulmonary fibrosis (IPF) is a fibrotic lung disease of unknown origin that is chronic, progressive, and eventually fatal. IPF mainly affects older adults, especially those in their sixth and seventh decades. Despite promising results with novel drugs such as pirfenidone and nintedanib, the mortality of patients with IPF remains high; median survival time is 2.5–3.5 years^1–3^. Furthermore, IPF disease progression is highly variable; while some IPF patients experience a reduction of symptoms over time, others are stable, experience slow worsening of respiratory symptoms or pulmonary function, or have acute exacerbation of symptoms, ultimately leading to death^1^. Therefore, clinicians usually face challenges in predicting the clinical course in newly diagnosed IPF patients. Precise prediction of clinical course is important for developing treatment plans and providing clinicians with accurate information that must be communicated to patients and medical teams^4^.

Previous studies used clinical factors (age, gender, smoking status, finger clubbing, dyspnea, 6-minute walking distance, and hospitalization), pulmonary function tests (PFTs), change in PFT, high-resolution computed tomography (HRCT) findings or scores, pulmonary hypertension, molecular biomarkers (metalloproteinase-7 and C-reactive protein [CRP]), and pathologic finding as variables in predictive models^5–11^. However, most of these predictive models are too complex to use and have not been validated externally.

In 2003, Wells *et al*.^12^ reported on their composite physiologic index (CPI), which was developed as a tool to reflect the morphologic extent of pulmonary fibrosis in IPF on computed tomography (CT) (*r* = 0.71, *r*^2^ = 0.51, *P* < 0.0005); it is calculated easily based on lung function parameters. CPI was a more powerful prognostic marker for mortality than either lung function or alveolar-arterial O_2_ gradient in surgically diagnosed IPF patients (*P* < 0.0005).

However, gender-age-physiology (GAP) stage, which based on the Fine-Gray competing risk models by Ley *et al*.^13^ in 2012, uses gender, age, and two pulmonary function results (percent predicted forced vital capacity [FVC] and percent predicted diffusing capacity of the lung for carbon monoxide [DL_CO]_). Ley *et al*. analyzed a relatively large cohort; they obtained a C-index of 69.3 in the derivation cohort (*n* = 228) and 68.7 in the validation cohort (*n* = 330).

The aim of this study was to validate and compare the predictive values of CPI and GAP stage models in the Korean population.

## Methods

### Patient selection

In this retrospective study, patients with newly diagnosed interstitial lung disease (ILD) were enrolled by the Korean Interstitial Lung Disease Research Group from January 1, 2003, through December 31, 2007^14^. The cut-off date was December 31, 2009. The diagnosis was performed by pulmonologists, radiologists, and pathologists at each hospital, and diagnosis was reconfirmed by the Scientific Committee at the Korean Academy of Tuberculosis and Respiratory Diseases. Fifty-four hospitals registered a total of 2,186 ILD patients. Patients with a condition other than IPF (*n* = 501) and patients with incomplete data (*n* = 423) were excluded from this study. We diagnosed IPF according to the previous international consensus statement by the American Thoracic Society (ATS), European Respiratory Society (ERS), and the American College of Chest Physicians^15,16^. Of 1,262 IPF patients, 430 patients lost to follow-up were also excluded from this study. As a result, a total of 832 patients with IPF were included in this study. The clinical data (age, sex, smoking, respiratory symptoms, diagnostic method, and mortality), radiologic findings (HRCT), laboratory data (arterial blood gas analysis, CRP, antinuclear antibody [ANA], and rheumatoid factor [RF]), and physiological data (PFT) were investigated. These data were saved in a web-based registry system (www.ild.or.kr). The mean follow-up period was 22.5 ± 16.3 months.

### Predictive model

CPI was calculated according to Wells *et al*.^12^: CPI parameters as follows: 91.0 – (0.65 × DL_CO_ percentage of the predicted value [% pred]) − (0.53 × FVC % pred) + (0.34 × forced expiratory volume [FEV_1_] % pred). Mura *et al*.^11^ showed that a CPI > 41.0 was significantly associated with 3-year survival in a prospective cohort (hazard ratio [HR] = 5.36, *P* = 0.0071), as well as in a retrospective cohort (HR = 4.20, *P* = 0.042). Based on their study, our patients were divided into two groups according to the calculated CPI value (≤41.0 and >41.0), and their characteristics were examined. GAP score was calculated according to Ley *et al*.^13^: gender (0–1 points), age (0–2 points), %FVC (0–2 points), and %DL_CO_ (0–3 points). However, the category “cannot perform DL_CO_ (3 points)” was not considered in the present study because of the retrospective nature of the study. GAP stage was determined based on the total GAP score: stage I (0–3 points), stage II (4–5 points), and stage III (6–8 points).

The primary objective of this study was to compare the predictive ability of CPI and GAP stage models for 1-year, 2-year, and 3-year mortality.

### Statistical analysis

Continuous variables were compared by *t*-test or analysis of variance (ANOVA) according to the number of groups, and these variables were presented as means ± standard deviation. Comparisons between GAP stages were performed with ANOVA, and post hoc analyses were conducted using Bonferroni’s correction. Categorical variables were analyzed by Pearson’s chi-square test, and these categorical variables were presented as frequency (*n*) and percentage (%). The predictive receiver operating characteristic (ROC) curves of 1-year, 2-year, and 3-year mortality were compared between CPI and GAP models. All statistics were analyzed with SPSS™ Version 22.0 (SPSS, Chicago, IL, USA). We considered an adjusted p-value less than 0.05 as statistically significant.

### Ethics statement

The Institutional Review Board (IRB) of Seoul National University Bundang Hospital approved this study protocol (IRB approval number: B-1709/420-102). Informed patient consent was waived due to the retrospective nature of our study. All methods were performed in accordance with the Declaration of Helsinki.

## Results

### Demographic characteristics

The baseline characteristics of the study population are presented in Table 1. The mean age of study population was 66.4 ± 9.3 years. Male gender was more prevalent than female gender (72.0% versus 23.0%). The most common respiratory symptom was dyspnea (68.0%), followed by cough (58.3%). However, 40 (4.8%) patients were asymptomatic. Mean smoking duration was 35.6 ± 12.7 years and mean smoking amount was 34.8 ± 20.0 pack-years. 73 patients (8.8%) had decreased lung function consistent with COPD (FEV_1_/FVC < 0.7 and age ≥40 years). A total of 380 (45.7%) patients were diagnosed by the surgical method, and 452 (54.3%) patients were diagnosed on the basis of clinical and radiographic criteria. Mean CPI was 38.6 ± 15.5; 460 (55.3%) patients had a CPI ≤ 41, and 372 (44.7%) patients had a CPI > 41 (Table 2). One-year mortality was 17.3%, 2-year mortality was 24.3%, and 3-year mortality was 29.7%. According to GAP, there were 536 (64.4%) stage I patients, 268 (32.2%) stage II patients, and 28 (3.4%) stage III patients.

**Table 1:** Baseline characteristics of IPF patients (*n* = 832).

Variable	
Age at diagnosis, yr	66.4 ± 9.3
Gender (F:M)	233 (28.0): 599 (72.0)
Duration of symptoms at diagnosis (month)	11.2 ± 20.9
Dyspnea of exertion(%)	566 (68.0)
Cough (%)	485 (58.3)
Sputum (%)	262 (31.5)
Hemoptysis (%	18 (2.2)
Chest pain (%	50 (6.0)
Asymptom (%)	40 (4.8)
*Smoking (%)	
Nonsmoker	280 (36.4)
Former	286 (37.2)
Current	203 (26.4)
Smoking duration (year)	35.6 ± 12.7
Smoking amounts (pack-year)	34.8 ± 20.0
Diagnostic method (%)	
Clinical-radiographic	452 (54.3)
Surgical	380 (45.7)
Outcome (%)	
Alive	559 (67.2)
Dead	273 (32.8)
CPI	38.6 ± 15.5
GAP stage (I/II/III)	536 (64.4)/268 (32.2)/28 (3.4)

Note: Values in parentheses are percentages.

^*^Smoking status were available for 769 patients.

CPI = 91.0 − (0.65 * DL_CO_ [%]) − (0.53 * FVC [%]) + (0.34 * FEV_1_ [%]).

ANA, antinuclear antibody; CPI, composite physiologic score; CRP, C-reactive protein; DL_CO_, diffusing capacity of the lung for carbon monoxide; FEV_1_, forced expiratory volume; FVC, forced vital capacity; GAP, (G, 0–1 point), age (A, 0–2 points), and 2 lung physiology variables (P, FVC and DL_CO_); RF, rheumatoid factor.

**Table 2:** Clinical, radiographic, and physiologic characteristics according to composite physiologic index (CPI).

	CPI ≤ 41 (*n* = 460)	CPI > 41 (*n* = 372)	*P*-value
Age	65.6 ± 9.0	67.3 ± 9.5	0.007
Gender (F:M)	116 (25.2)/344 (74.8)	117 (31.5)/255 (68.5)	0.046
Pulmonary function test			
FVC (%)	84.1 ± 17.3	69.2 ± 16.2	<0.001
FEV_1_ (%)	92.9 ± 18.6	81.7 ± 19.4	<0.001
TLC (%)	89.8 ± 18.9	74.5 ± 16.8	<0.001
DL_CO_ (%)	77.0 ± 17.6	46.7 ± 11.8	<0.001
Resting PaO_2_ mm Hg	86.5 ± 22.5	78.8 ± 20.0	<0.001
Resting PaCO_2_ mm Hg	38.4 ± 7.8	36.2 ± 6.9	0.001
Radiologic finding			
Reticular pattern	298 (68.0)	231 (68.5)	0.880
Honeycombing change	323 (74.9)	277 (78.7)	0.217
Ground glass opacities	279 (64.3)	206 (63.4)	0.798
Nodular lesions	94 (23.2)	70 (22.8)	0.898
GAP stage (I/II/III)	388/72/0	148/196/28	<0.001
Death (%)	112 (24.3)	161 (43.3)	<0.001

CPI, composite physiologic score; CRP, C-reactive protein; DL_CO_, diffusing capacity of the lung for carbon monoxide; FEV_1_, forced expiratory volume; FVC, forced vital capacity; GAP, (G, 0–1 point), age (A, 0–2 points), and 2 lung physiology variables (P, FVC and DL_CO_); PaO_2_, arterial oxygen tension; PaCO_2_, arterial carbon dioxide tension; TLC, total lung capacity.

Tables 2 and 3 show the clinical, radiologic, and physiologic characteristics according to CPI and GAP stage. The elevated CPI group (CPI > 41) was significantly associated with aging, low lung function, and low arterial oxygen tension (PaO_2_) (similar to advanced GAP stage). However, male sex was significantly predominant in the group with CPI ≤ 41 than in the group with CPI > 41. Radiologic findings were not significantly different between the two groups (CPI ≤ 41 and CPI > 41).

**Table 3:** Clinical, radiographic, and physiologic characteristics according to GAP stage.

	GAP stage I (n = 536)	GAP stage II (n = 268)	GAP stage III (n = 28)	*P*-value
Age	63.6 ± 9.1	71.4 ± 7.4	71.8 ± 4.5	<0.001
Gender (F:M)	194 (36.2): 342 (63.8)	38 (14.2): 230 (85.8)	1 (3.6): 27 (96.4)	<0.001
Pulmonary function test				
FVC (%)	83.0 ± 17.2	68.8 ± 16.0	55.9 ± 12.6	<0.001
FEV_1_ (%)	92.6 ± 19.1	81.0 ± 17.8	64.4 ± 13.6	<0.001
TLC (%)	86.6 ± 19.2	77.9 ± 18.1	69.9 ± 25.8	<0.001
DL_CO_ (%)	69.8 ± 18.9	54.3 ± 20.6	30.2 ± 11.4	<0.001
Resting PaO_2_ mm Hg	86.5 ± 21.7	77.3 ± 20.9	71.2 ± 12.6	<0.001
Resting PaCO_2_ mm Hg	38.3 ± 7.7	35.8 ± 7.0	36.1 ± 6.9	0.002
Radiologic finding				
Reticular pattern	353 (69.2)	162 (67.2)	14 (58.3)	0.490
Honeycombing change	372 (73.8)	206 (81.7)	22 (81.5)	0.043
Ground glass opacities	331 (65.3)	136 (59.1)	18 (81.8)	0.056
Nodular lesions	116 (24.1)	43 (20.5)	5 (23.8)	0.577
CPI	33.2 ± 13.5	46.8 ± 13.4	63.7 ± 7.4	<0.001
Death (%)	139 (25.9)	118 (44.0)	16 (57.1)	<0.001

^**‡**^The following post hoc comparisons were significant at the *P* = 0.05 level; all other comparisons were non-significant: GAP stage I versus GAP stage II, and GAP stage III (age); GAP stage I versus GAP stage II and GAP stage III, and GAP stage II versus GAP stage III (FVC [%]); GAP stage I versus GAP stage II and GAP stage III, and GAP stage II versus GAP stage III (FEV_1_ [%]); GAP stage I versus GAP stage II and GAP stage III (TLC [%]); GAP stage I versus GAP stage II and GAP stage III, and GAP stage II versus GAP stage III (DL_CO_ [%]); GAP stage I versus GAP stage II, and GAP stage III (PaO_2_); GAP stage I versus GAP stage II (PaCO_2_); GAP stage I versus GAP stage II and GAP stage III, and GAP stage II versus GAP stage III (CPI).

CPI = 91.0 − (0.65 * DL_CO_ [%]) − (0.53 * FVC [%]) + (0.34 * FEV_1_ [%]).

CPI, composite physiologic score; CRP, C-reactive protein; FEV_1_ = forced expiratory volume; FVC = forced vital capacity; TLC = total lung capacity; DL_CO_ = diffusing capacity of the lung for carbon monoxide; GAP, (G, 0–1 point), age (A, 0–2 points), and 2 lung physiology variables (P, FVC and DL_CO_); PaO_2_ = arterial oxygen tension; PaCO_2_ = arterial carbon dioxide tension.

Advanced GAP stage was significantly associated with aging, reduced lung function, low PaO_2_, and higher mortality rate. CPI were significantly increased in patients with advanced GAP stage (*P* < 0.001). However, in radiologic findings, only the percentage of honeycombing was significantly different among GAP stages (*P* = 0.043).

### Survival according to predictive models

**Figure 1:**
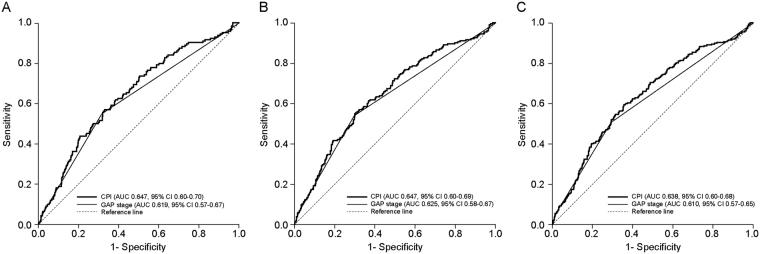
Receiver operator characteristic (ROC) curves of GAP stage and CPI to predict mortality in all IPF patients (*n* = 832). (**A**) 1-year mortality, (**B**) 2-year mortality, and (**C**) 3-year mortality. All predictive models were significantly robust to predict mortality. CPI model was more accurate than GAP stage to predict 1-year mortality (p = 0.301), 2-year mortality (p = 0.349), and 3-year mortality (p = 0.220), but it did not significant. Note: The straight line in the middle is the reference line. AUC, area under the curve; CPI, composite physiologic index; GAP, (G, 0–1 point), age (A, 0–2 points), and 2 lung physiology variables (P, FVC and DL_CO_); IPF, idiopathic pulmonary fibrosis; ROC, receiver operator characteristic.

CPI and GAP stage significantly predicted disease progression according to the Cox proportional hazard model (Table 4). The HR increased with increased CPI score (*P* < 0.001; HR, 1.025; 95% confidence interval [CI], 1.017–1.034), GAP score (*P* < 0.001; HR, 1.332; 95% CI, 1.222–1.451), and with advanced GAP stage. The predictive value of CPI and GAP stage at 1-year, 2-year, and 3-year mortality was assessed by ROC curve analysis. Each model showed significant predictive capacity at all time points. ROC curves for all patients with IPF are shown in Fig. 1. The area under the curve (AUC) for 1-year mortality was 0.619 for GAP stage and 0.647 for CPI. The AUC for 2-year mortality was 0.625 for GAP stage and 0.647 for CPI. The AUC for 3-year mortality decreased; it was 0.610 for GAP stage and 0.639 for CPI. CPI predicted survival more accurately than did GAP stage for all time points modelled, but the difference in AUC was not statistically significant (Table S1 [Supplementary Information]). Well *et al*. reported that CPI had the greatest prognostic significance compared with lung function results or PaO_2_ in biopsy-proven IPF. Therefore, we investigated the GAP stage and CPI in surgically diagnosed IPF patients (Fig. S1 and Table S1 [Supplementary Information]). In surgically diagnosed IPF patients, the AUC for 1-year mortality was 0.622 for GAP stage and 0.673 for CPI. The AUC for 2-year mortality was 0.624, and 0.674, respectively. The AUC for 3-year mortality was 0.602, and 0.667, respectively. Generally, CPI was a more accurate predictor of mortality than GAP stage. In clinically diagnosed IPF patients, the two predictive models showed statistically significant AUC values, but no significant differences were found between them (Fig. S2 [Supplementary Information]). The predictive value for outcome with AUC was under 0.62 in clinically diagnosed IPF patients, which was lower than the value in surgically diagnosed IPF patients.

**Table 4:** Univariate analysis of survival in idiopathic pulmonary fibrosis using Cox proportional hazard model (3-year survival).

Variables	Number of Patients	Relative hazard rate	95% CI	*P*-value
CPI	832	1.025	1.017–1.034	<0.001
GAP score	832	1.332	1.222–1.451	<0.001
GAP stage				<0.001
Stage I	536			
Stage II	268	1.954	1.528–2.498	<0.001
Stage III	28	2.518	1.500–4.225	<0.001

CPI = 91.0 − (0.65 * DL_CO_ [%]) − (0.53 * FVC [%]) + (0.34 * FEV_1_ [%]).

CPI, composite physiologic score; FEV_1_ = forced expiratory volume; FVC = forced vital capacity; GAP, (G, 0–1 point), age (A, 0–2 points), and 2 lung physiology variables (P, FVC and DL_CO_).

## Discussion

CPI and GAP stage are easy to use and can be calculated in-office during the initial visit and thereafter during follow-up visits. CPI can be calculated from PFT results, and the GAP stage calculation requires only age and gender in addition to PFT results^12,13^. In this retrospective study, we compared the predictive value of CPI and GAP stages. Both were effective for predicting mortality. However, both models had a limited capability to provide accurate prognoses for IPF patients.

As mentioned previously, predictive models of IPF prognosis rely on numerous clinical factors, physiologic parameters, radiologic features, biomarkers, and pathologic findings^5–11,17,18^. Most models developed to date are complex. In this study, we demonstrated that two simple-to-use models (GAP stage and CPI) have important predictive values. CPI was developed in a British study and has the advantage of relying on PFT data to predict IPF prognosis. Additionally, CPI was developed by considering the severity of emphysema, which could lead to overestimation of lung function^19^. However, CPI also has disadvantages: it does not consider clinical data such as age, gender, smoking history, presence of desaturation, or 6-minute walk distance. Furthermore, the value of CPI as a prognostic model is not well-studied. In the original publication, this issue had been determined only in 32 histologically proven subjects with the usual interstitial pneumonia pattern, leading to the hypothesis that CPI may be useful as a prognostic marker^12^. In this study, we tested CPI as a predictive model for 1-, 2-, and 3-year mortality with a relatively large cohort (*n* = 832); CPI was effective for prediction of the outcomes of IPF patients (*P* < 0.001, Table 4), and the AUC was approximately 0.61–0.65 in all patients. Additionally, CPI was more accurate than GAP stage, regardless of time of year or diagnostic method.

GAP staging, which was developed in a study in the United States and Italy, uses the clinical data of gender and age, and two sets of physiological data. However, it does not consider HRCT findings. Coexistent emphysema can be a confounding factor due to increasing %FVC, which could cause clinicians to render a good prognosis with a low GAP score^20^. In our study, although GAP stage showed a lower AUC value than CPI in predicting 1-, 2-, and 3- year mortality, it exhibited significance in the prediction of mortality (Table 4). Similarly, Sharp *et al*.^21^ reported that the AUC is lower in the GAP staging system than the CPI at both 12 months and 24 months. Additionally, age showed higher AUC than the GAP staging system at 12 months in their study. These findings could mean that gender is not a strong predictor of mortality. Furthermore, the pathophysiology of IPF is so complex that even multi-dimensional approaches might fail to predict the prognosis with sufficient accuracy^22^. Additional assessment of changes in functional lung capacity over time or biomarkers could be helpful in improving predictability^23^.

In our study, advanced GAP stage was understandably significantly associated with aging, male gender, and poor lung function. However, patients with elevated CPI (CPI > 41) showed significantly low predominance of male gender compared with patients with low CPI (CPI ≤ 41). This could mean that gender has a lesser effect on survival compared with age, HRCT, or PFT. Some studies have shown that gender affects survival, but others have reported no association between the two factors^6,10,24–26^. King *et al*.^6^ showed there was no significant gender difference on median survival using Kaplan–Meier analysis (*P* = 0.15; men, 30.0 months; CI: 19.1–44.3; women, 39.3 months; CI: 19.1–44.3). On the contrary, Flaherty *et al*.^27^ showed that female gender was protective in IIP, and when surgically diagnosed IPF patients were added to the analysis, female gender showed a significantly low HR. Douglas *et al*.^28^ also reported that male gender was significantly associated with a worse outcome.

CPI was more precise in surgically proven IPF patients in our study (Figs S1, S2). Generally, physicians are unwilling to biopsy IPF patients who are in medically poor condition^29^. As a result, surgically diagnosed IPF patients showed a significantly lower GAP index and lower CPI level than clinically diagnosed IPF patients (Supplementary Table 1). These results may mean that the predictive capabilities of CPI and the GAP stage system are more accurate in early IPF patients than in advanced IPF patients, and these models are not very reliable for longitudinal assessment. Recently, another study demonstrated that previous multi-dimensional indices for IPF were not more powerful as prognostic markers than clinical or physiologic parameters; DL_CO_ is the more powerful prognostic marker on longitudinal follow-up than CPI or GAP stage^21^. These could mean that although these two models were created taking into consideration age, gender, lung function, and CT findings, these data were not sufficient to predict the outcome due to the heterogeneity of IPF. Further risk assessments (e.g., 6-minute walking test, oxygen demand, hospitalization due to respiratory problem, pulmonary hypertension, acute exacerbation, or lung cancer), should be considered for a more precise prediction of prognosis in IPF, especially for longitudinal follow-up.

Although GAP stage and CPI models showed significant capability in prediction and association with relative risk (Table 4) on mortality, the AUC values of the CPI and GAP stage were low in this study, especially those for 3-year mortality in GAP stage. This may be due to ethnic differences, complexity of the pathophysiology of IPF itself, and uneven distribution of population in the GAP stage (low numbers in GAP stage III). Kim *et al*.^26^ demonstrated that the GAP model failed to predict 3-year mortality with Korean patients. Additionally, 430 (34.1%) patients were lost to follow-up and excluded in this study. This sizeable patient set could have resulted in selection bias, and may have caused a decrease in the predictive capabilities of the two models. Furthermore, this study may have included some patients with combined pulmonary fibrosis and emphysema (CPFE). Although the prognosis between IPF and CPFE is still unclear, patients with CPFE could affect the GAP model^30–32^.

This study had some limitations. First, our patients were diagnosed based on the ATS/ERS criteria published in 2002^15,16^. Therefore, IPF patients, who were diagnosed based on 2011 ATS/ERS/JRS/ALAT guidelines could have a different prognosis^1^. However, GAP stage and CPI were also first tested in patients who were diagnosed using the 2002 guidelines. Second, we could not correlate HRCT finding with CPI score. Although CPI was also elevated in patients with advanced GAP stage in a previous study from Japan, CPI has not yet been validated in Asian patients^33^. Third, our study is retrospective in nature, so we excluded patients who did not undergo DL_CO_ testing, many of whom may have been included in the “cannot perform DL_CO_” category in the GAP stage model. As a result, the GAP stage III group was small compared with the others, which could result in selection bias.

In conclusion, both GAP stage and CPI showed significant capabilities to predict mortality, and CPI was more accurate than GAP stage in predicting mortality at 1, 2, and 3 years. However, the complexity of IPF and the inconsistencies in physiologic and clinical parameters limit the capability of both models to provide accurate prognoses for IPF patients. Further large-scale prospective studies are needed to investigate a more accurate predictive model.

## Electronic supplementary material

Supplementary Information

## References

[1] Raghu G (2011). An official ATS/ERS/JRS/ALAT statement: idiopathic pulmonary fibrosis: evidence-based guidelines for diagnosis and management. Am J Respir Crit Care Med.

[2] Raghu G (2015). An Official ATS/ERS/JRS/ALAT Clinical Practice Guideline: Treatment of Idiopathic Pulmonary Fibrosis. An Update of the 2011 Clinical Practice Guideline. Am J Respir Crit Care Med.

[3] Ley B, Collard HR, King TE (2011). Clinical course and prediction of survival in idiopathic pulmonary fibrosis. Am J Respir Crit Care Med.

[4] Rozanski C, Mura M (2014). Multi-Dimensional Indeces to Stage Idiopathic Pulmonary Fibrosis: A Systematic Review. Sarcoidosis Vasc Dif.

[5] Gay SE (1998). Idiopathic pulmonary fibrosis - Predicting response to therapy and survival. Am J Resp Crit Care.

[6] King TE, Tooze JA, Schwarz MI, Brown KR, Cherniack RM (2001). Predicting survival in idiopathic pulmonary fibrosis: Scoring system and survival model. Am J Resp Crit Care.

[7] Mogulkoc N (2001). Pulmonary function in idiopathic pulmonary fibrosis and referral for lung transplantation. Am J Resp Crit Care.

[8] du Bois RM (2011). Ascertainment of Individual Risk of Mortality for Patients with Idiopathic Pulmonary Fibrosis. Am J Resp Crit Care.

[9] Richards TJ (2012). Peripheral Blood Proteins Predict Mortality in Idiopathic Pulmonary Fibrosis. Am J Resp Crit Care.

[10] Lee SH (2011). Prognostic Factors for Idiopathic Pulmonary Fibrosis: Clinical, Physiologic, Pathologic, and Molecular Aspects. Sarcoidosis Vasc Dif.

[11] Mura M (2012). Predicting survival in newly diagnosed idiopathic pulmonary fibrosis: a 3-year prospective study. Eur Respir J.

[12] Wells AU (2003). Idiopathic pulmonary fibrosis - A composite physiologic index derived from disease extent observed by computed tomography. Am J Resp Crit Care.

[13] Ley B (2012). A Multidimensional Index and Staging System for Idiopathic Pulmonary Fibrosis. Ann Intern Med.

[14] Lee, S. H. *et al*. Predicting survival of patients with idiopathic pulmonary fibrosis using GAP score: a nationwide cohort study. *Resp Res* 1**7**, 10.1186/s12931-016-0454-0 (2016). 10.1186/s12931-016-0454-0 PMC5069824 27756398

[15] American Thoracic Society (2000). Idiopathic pulmonary fibrosis: diagnosis and treatment. International consensus statement. American Thoracic Society (ATS), and the European Respiratory Society (ERS). Am J Respir Crit Care Med.

[16] Agusti C (2002). American Thoracic Society/European Respiratory Society International Multidisciplinary Consensus Classification of the Idiopathic Interstitial Pneumonias (vol 165, pg 277, 2002). Am J Resp Crit Care.

[17] Barlo NP, van Moorsel CH, van den Bosch JM, Grutters JC (2010). Predicting prognosis in idiopathic pulmonary fibrosis. Sarcoidosis Vasc Diffuse Lung Dis.

[18] Vainshelboim B, Oliveira J, Fox BD, Kramer MR (2016). The Prognostic Role of Ventilatory Inefficiency and Exercise Capacity in Idiopathic Pulmonary Fibrosis. Respir Care.

[19] Lynch JP (2016). Idiopathic Pulmonary Fibrosis: Epidemiology, Clinical Features, Prognosis, and Management. Semin Respir Crit Care Med.

[20] Wells AU (1997). Lone cryptogenic fibrosing alveolitis: a functional-morphologic correlation based on extent of disease on thin-section computed tomography. Am J Respir Crit Care Med.

[21] Sharp, C., Adamali, H. I. & Millar, A. B. A comparison of published multidimensional indices to predict outcome in idiopathic pulmonary fibrosis. *ERJ Open Res* **3**, 10.1183/23120541.00096-2016 (2017). 10.1183/23120541.00096-2016 PMC5349096 28326312

[22] Liu YM, Nepali K, Liou JP (2017). Idiopathic Pulmonary Fibrosis: Current Status, Recent Progress, and Emerging Targets. J Med Chem.

[23] Rozanski C, Mura M (2014). Multi-dimensional indices to stage idiopathic pulmonary fibrosis: a systematic review. Sarcoidosis Vasc Diffuse Lung Dis.

[24] Flaherty KR (2003). Prognostic implications of physiologic and radiographic changes in idiopathic interstitial pneumonia. Am J Respir Crit Care Med.

[25] Nadrous HF (2005). Pulmonary hypertension in patients with idiopathic pulmonary fibrosis. Chest.

[26] Enomoto N (2006). Quantitative analysis of fibroblastic foci in usual interstitial pneumonia. Chest.

[27] Flaherty KR (2002). Clinical significance of histological classification of idiopathic interstitial pneumonia. Eur Respir J.

[28] Douglas WW, Ryu JH, Schroeder DR (2000). Idiopathic pulmonary fibrosis: Impact of oxygen and colchicine, prednisone, or no therapy on survival. Am J Respir Crit Care Med.

[29] Lee, S. H. *et al*. Comparisons of Prognosis between Surgically and Clinically Diagnosed Idiopathic Pulmonary Fibrosis Using Gap Model A Korean National Cohort Study. *Medicine* **95**, 10.1097/MD.0000000000003105 (2016). 10.1097/MD.0000000000003105 PMC4839935 26986154

[30] Lin H, Jiang S (2015). Combined pulmonary fibrosis and emphysema (CPFE): an entity different from emphysema or pulmonary fibrosis alone. J Thorac Dis.

[31] Mejia M (2009). Idiopathic pulmonary fibrosis and emphysema: decreased survival associated with severe pulmonary arterial hypertension. Chest.

[32] Ryerson CJ (2013). Clinical features and outcomes in combined pulmonary fibrosis and emphysema in idiopathic pulmonary fibrosis. Chest.

[33] Kishaba T (2015). Clinical characteristics of idiopathic pulmonary fibrosis patients with gender, age, and physiology staging at Okinawa Chubu Hospital. J Thorac Dis.

