# Supplementary material for: Comparison of CPI and GAP models in patients with idiopathic pulmonary fibrosis: a nationwide cohort study
Source: Sci Rep. 2018 Mar 19;8:4784. doi: 10.1038/s41598-018-23073-3 (PMC5859191; doi:10.1038/s41598-018-23073-3)
Supplement: Supplementary file 1 — Supplementary Information [file 41598_2018_23073_MOESM1_ESM.doc]

**Comparison of CPI and GAP models in patients with idiopathic pulmonary fibrosis: a nationwide cohort study**

Sang Hoon Lee, Jong Sun Park, Song Yee Kim, Dong Soon Kim, Young Whan Kim, Man Pyo Chung, Soo Taek Uh, Choon Sik Park, Sung Woo Park, Sung Hwan Jeong, Yong Bum Park, Hong Lyeol Lee, Jong Wook Shin, Eun Joo Lee, Jin Hwa Lee, Yangin Jegal, Hyun Kyung Lee, Yong Hyun Kim, Jin Woo Song, Moo Suk Park

**Table S1. Receiver operating characteristic (ROC) data of GAP stage and CPI according to diagnostic method and at each time point**

|  | | CPI | | | |  | GAP | | | |
| --- | --- | --- | --- | --- | --- | --- | --- | --- | --- | --- |
|  | | sensitivity | specificity | AUC | p-value |  | sensitivity | specificity | AUC | p-value |
| All | |  |  |  |  |  |  |  |  |  |
|  | 1 year | 56.9 | 67.0 | 0.647 | <0.001 |  | 54.9 | 68.5 | 0.619 | <0.001 |
|  | 2 year | 55.4 | 69.5 | 0.647 | <0.001 |  | 54.0 | 70.3 | 0.625 | <0.001 |
|  | 3 year | 59.5 | 64.3 | 0.638 | <0.001 |  | 50.6 | 70.8 | 0.610 | <0.001 |
| Surgical | |  |  |  |  |  |  |  |  |  |
|  | 1 year | 81.0 | 47.8 | 0.673 | <0.001 |  | 37.9 | 86.0 | 0.622 | 0.004 |
|  | 2 year | 73.3 | 57.4 | 0.674 | <0.001 |  | 37.3 | 87.2 | 0.624 | 0.001 |
|  | 3 year | 71.4 | 58.5 | 0.667 | <0.001 |  | 33.0 | 87.2 | 0.602 | 0.004 |
| Clinical | |  |  |  |  |  |  |  |  |  |
|  | 1 year | 51.2 | 72.4 | 0.619 | <0.001 |  | 66.3 | 53.0 | 0.599 | 0.005 |
|  | 2 year | 59.1 | 62.5 | 0.609 | <0.001 |  | 63.8 | 54.5 | 0.597 | 0.001 |
|  | 3 year | 56.4 | 63.2 | 0.594 | <0.001 |  | 60.9 | 54.7 | 0.585 | 0.003 |

CPI = 91.0 – (0.65 * DL_CO_ [%]) – (0.53 * FVC [%]) + (0.34 * FEV_1_ [%])

CPI, composite physiologic score; FEV_1_ = forced expiratory volume; FVC = forced vital capacity; GAP, (G, 0-1 point), age (A, 0–2 points), and 2 lung physiology variables (P, FVC and DL_CO_); AUC, area under the curve

**Figure S1.** Receiver operator characteristic (ROC) curves of GAP stage and CPI to predict mortality in IPF patients diagnosed surgically (*n* = 380). (A) 1-year mortality, (B) 2-year mortality, and (C) 3-year mortality.


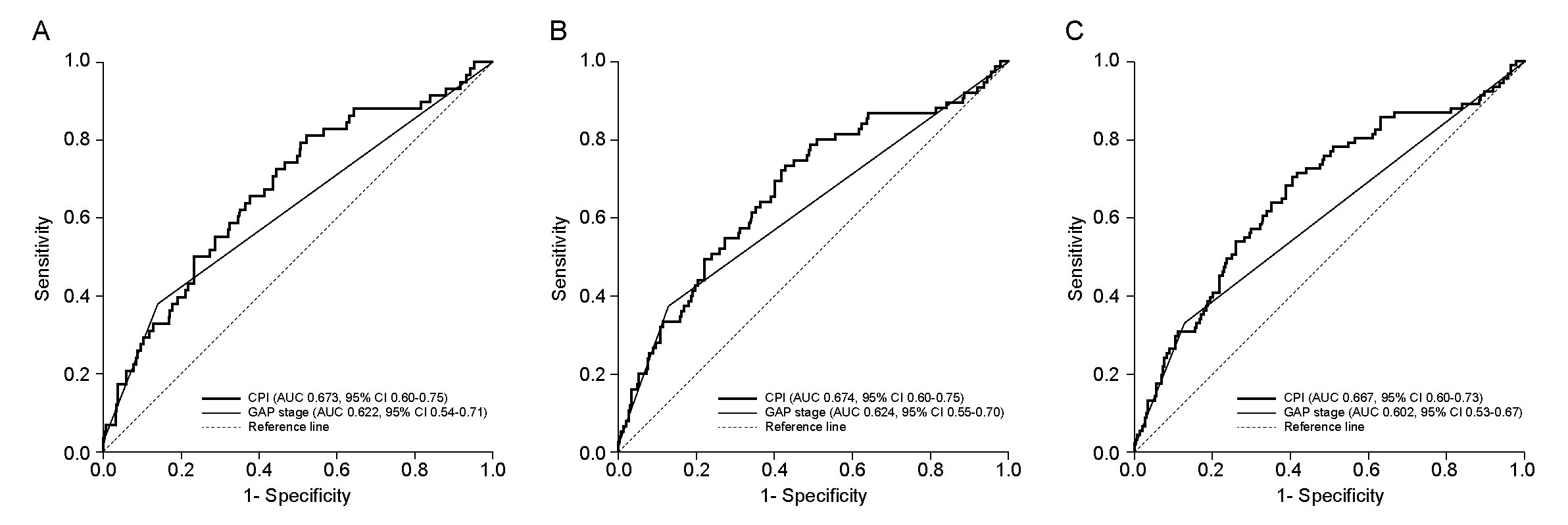


All predictive models showed significant association with predicting mortality. CPI model was more accurate than GAP stage to predict 1-year mortality (*P* = 0.279), 2-year mortality (*P* = 0.239), and 3-year mortality (*P* = 0.100), but it was not significant.

AUC, area under the curve; CPI, composite physiologic index; GAP, (G, 0-1 point), age (A, 0–2 points), and 2 lung physiology variables (P, FVC and DL_CO_)

**Figure S2.** Receiver operator characteristic (ROC) curves of GAP stage and CPI to predict mortality in idiopathic pulmonary fibrosis patients diagnosed clinically (n = 452). (A) 1-year mortality, (B) 2-year mortality, and (C) 3-year mortality.

**
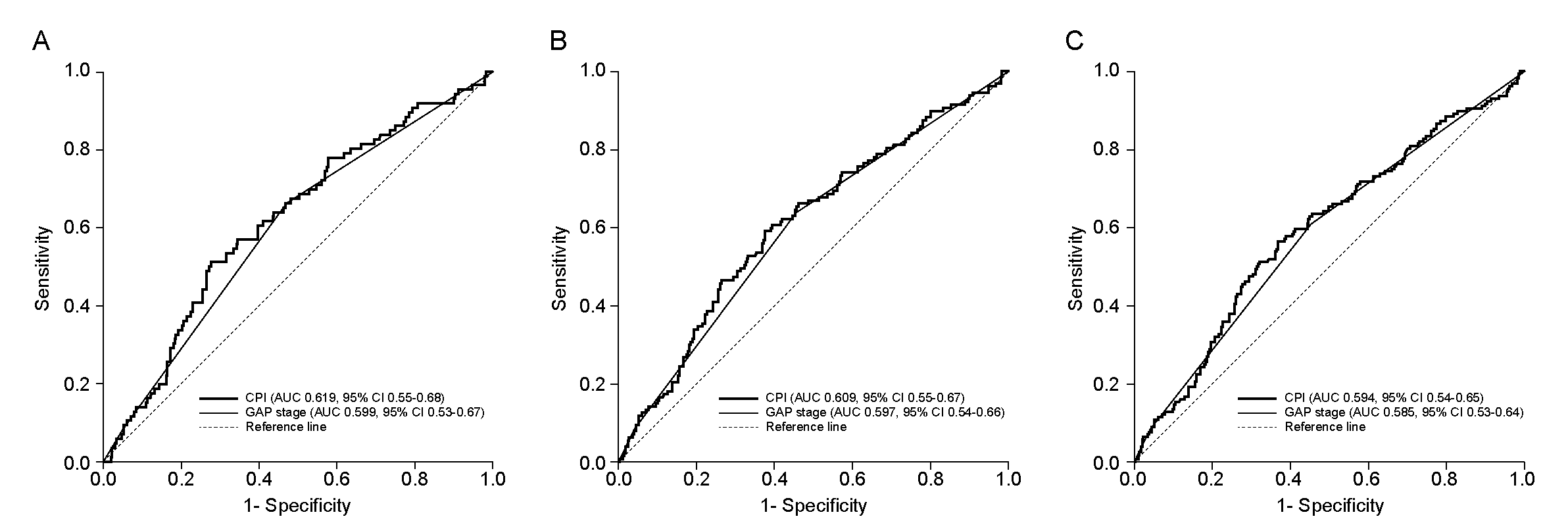
**

CPI model was more accurate than GAP stage to predict 1-year mortality (*P* = 0.574), 2-year mortality (*P* = 0.699), and 3-year mortality (*P* = 0.733), but it was not significant.

AUC, area under the curve; CPI, Composite physiologic index; GAP, (G, 0-1 point), age (A, 0–2 points), and 2 lung physiology variables (P, FVC and DL_CO_)
